# Winners, Losers, Insiders, and Outsiders: Comparing Hierometer and Sociometer Theories of Self-Regard

**DOI:** 10.3389/fpsyg.2016.00334

**Published:** 2016-03-30

**Authors:** Nikhila Mahadevan, Aiden P. Gregg, Constantine Sedikides, Wendy G. de Waal-Andrews

**Affiliations:** ^1^Centre for Research on Self and Identity, School of Psychology, University of SouthamptonSouthampton, UK; ^2^Department of Social Psychology, Tilburg UniversityTilburg, Netherlands

**Keywords:** hierometer theory, sociometer theory, status, inclusion, self-regard, self-esteem, narcissism, assertiveness

## Abstract

What evolutionary function does self-regard serve? Hierometer theory, introduced here, provides one answer: it helps individuals navigate status hierarchies, which feature zero-sum contests that can be lost as well as won. In particular, self-regard tracks social status to regulate behavioral assertiveness, augmenting or diminishing it to optimize performance in such contests. Hierometer theory also offers a conceptual counterpoint that helps resolve ambiguities in sociometer theory, which offers a complementary account of self-regard’s evolutionary function. In two large-scale cross-sectional studies, we operationalized theoretically relevant variables at three distinct levels of analysis, namely, *social* (relations: status, inclusion), *psychological* (self-regard: self-esteem, narcissism), and *behavioral* (strategy: assertiveness, affiliativeness). Correlational and mediational analyses consistently supported hierometer theory, but offered only mixed support for sociometer theory, including when controlling for confounding constructs (anxiety, depression). We interpret our results in terms of a broader agency-communion framework.

## Introduction

The human self is notoriously hard to pin down. Frustrated, some modern philosophers have questioned its very existence (e.g., Metzinger, 2003). Yet the self still somehow matters: people want their self, whatever it is, to be “good.” In other words, they want to achieve positive *self-regard* (Sedikides and Gregg, 2008).^1^ And understandably so: positive self-regard provokes pleasant feelings whereas negative self-regard provokes painful ones (Gregg et al., 2011).

But why should such a hedonic contingency exist in the first place? Otherwise put, what ultimate *function* might self-regard serve? Various theories have addressed this question. For example, *terror management theory* (Pyszczynski et al., 2004) claims that the purpose of self-regard is to buffer the potentially paralyzing terror that humans experience on contemplating death. Alternatively, *self-determination theory* (Deci and Ryan, 2000) claims that self-regard does not serve any specific purpose, but that its character depends on whether or not the social environment satisfies fundamental needs.

However, perhaps the leading contemporary account of the function of self-regard is provided by *sociometer theory* (Leary et al., 1995). This posits that self-regard operates as part of an adaptive psychological system that fitted ancestral human beings to social living. In this paper, both to address ambiguities in sociometer theory, and to make new intellectual headway, we introduce a novel alternative account of self-regard’s evolutionary function: *hierometer theory*. We outline the fundamentals about both hierometer theory and sociometer theory and review the empirical evidence for them. We then report on a research program evaluating whether and to what extent patterns of association between variables at three different levels of analysis (social, psychological, behavioral) tend to confirm or infirm each of the theories.

### Sociometer Theory: Theoretical Outline

Sociometer theory starts from the premise that human beings have a fundamental *need to belong* (Baumeister and Leary, 1995). Satisfying this need is advantageous: group members, when cooperating, afford one another significant opportunities for mutual gain (von Mises, 1963; Nowak and Highfield, 2011; Wilson, 2012). Accordingly, if individuals are excluded from key social networks, their prospects for surviving and reproducing are impaired. It is therefore plausible to hypothesize that a dedicated psychological system evolved to encourage *social acceptance* (Leary et al., 1995). Such a system would serve two complementary functions, which we here label *indicative* and *imperative*.

The indicative function would be to *track an individual’s level of social acceptance* (or, more anthropomorphically, to “monitor” it). Logically, to enjoy the benefits that accrue from mutually supportive relationships, some level of social acceptance is required. To the extent that individuals achieve such social acceptance, they will enjoy higher *relational value*, defined as the extent to which (they believe that) other group members consider it worthwhile to associate with them (Leary, 1999, 2005). Accordingly, a system designed to track one’s social acceptance—in particular, to pick up on interpersonal cues that might portend lower relational value—would be useful. If such cues were detected, the relevant imperative function of the system would be triggered. It would *propel an individual to act so as to meet a minimal level of social acceptance*. In particular, if social acceptance diverged from this minimum, then the system would seek to reduce this divergence, by prompting an individual to engage in remedial prosocial behaviors.

Sociometer theory contends that self-regard—more specifically, *self-esteem*—serves both these functions. Initially, it tracks levels of social acceptance, by rising and falling in tandem with them (i.e., its indicative function). A fall in self-esteem sends an intrapsychic signal that one’s social acceptance has dropped, perhaps critically. This signal, if sufficiently urgent, motivates individuals to act in ways that restore or reinforce social acceptance (i.e., its imperative function). Thus, the sociometer system is said to operate rather like the digestive system: an empty stomach (cf. low social acceptance) leads to unpleasant hunger pangs (cf. low self-esteem) that prompt one to fill one’s stomach by ingesting food (cf. regain social acceptance by acting prosocially). Fundamentally, then, sociometer theory is a theory of *insiders* and *outsiders*.

### Sociometer Theory: Empirical Evidence

Sociometer theory enjoys empirical support. In particular, there is good evidence that self-esteem serves its hypothesized indicative function, and mixed evidence that it serves its hypothesized imperative function.

### Indicative Function: Tracking Social Acceptance

Consistent with sociometer theory, self-esteem covaries with perceptions of social acceptance and with levels of social connectivity (Leary et al., 1995; Leary et al., 2001, Study 3; Denissen et al., 2008, Study 5). Self-esteem is also higher to the extent that individuals believe that they possess traits liable to promote social acceptance or social approval (MacDonald et al., 2003; Anthony et al., 2007a). In addition, anticipated levels of self-esteem—after performing various actions, or after various events occur—covary with how individuals expect others will react (Leary et al., 1995, Studies 1 and 2; Koch and Shepperd, 2008, Studies 3 and 4). Finally, receiving rejection feedback, real or imagined, from personal or impersonal sources, lowers self-esteem (Leary et al., 1998; Zadro et al., 2004; but see Blackhart et al., 2009; Bernstein et al., 2013).

### Imperative Function: Prompting Affiliative Behavior

Consistent with sociometer theory, socially excluded individuals evaluate others more favorably, express a stronger desire to work with them, and report a greater interest in making new friends (Maner et al., 2007). They also conform more to collective opinion (Williams et al., 2000) and tailor their purchases toward products that promote inclusion (Mead et al., 2011). In addition, individuals with low self-esteem opt to join groups only when acceptance in them is guaranteed or undemanding, consistent with their being unwilling to risk further rejection (Haupt and Leary, 1997; Anthony et al., 2007b).

Contrary to sociometer theory, however, social exclusion leads individuals to derogate those who exclude them (Leary et al., 1995; Bourgeois and Leary, 2001), reduces their empathy for and willingness to help others generally (Twenge et al., 2007), and increases their overall levels of hostility and aggression (Twenge et al., 2001; Buckley et al., 2004). Moreover, individuals with low self-esteem are relatively less likely to initiate social interactions and new relationships (Baumeister et al., 2003; Anthony et al., 2007b). Such anti-social reactions seem unlikely to promote social acceptance or augment relational value.

### Hierometer Theory: Theoretical Outline

**FIGURE 1:**
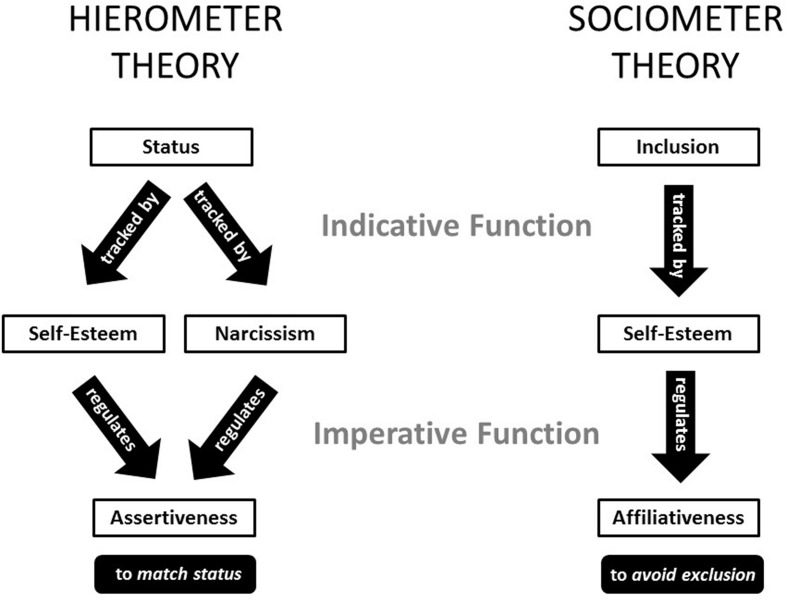
**A side-by-side illustration of the hypothesized dynamics of hierometer theory and (the original version of) sociometer theory**.

Like sociometer theory, hierometer theory proposes that self-regard serves an evolutionary function. Unlike sociometer theory, it proposes that this function is to *navigate status hierarchies*. Specifically, hierometer theory proposes that self-regard operates both indicatively—by tracking levels of social status—and imperatively—by regulating levels of status pursuit (**Figure 1**).

Status hierarchies are pervasive (Sidanius and Pratto, 1999; Sapolsky, 2005). They exist in both humans and animals, in simple and complex societies, and in formal and informal groups (Mazur, 1985; Anderson et al., 2001, 2006). Moreover, these hierarchies matter: higher-status individuals enjoy better health and well-being (Marmot, 2004; Sapolsky, 2005), a wider choice of romantic partners (Betzig, 1986), and greater reproductive success (von Rueden et al., 2008, 2011).

Given the significance of status hierarchies, a dedicated psychological system is liable to have evolved to help individuals navigate them successfully (cf. Gilbert, 2000; Sloman, 2008). Certainly, high status is desirable given the benefits it brings, and the desire to achieve it is often considered fundamental (Barkow, 1975, 1980; Frank, 1985; Marmot, 2004; Anderson et al., 2015). Nonetheless, the indiscriminate pursuit of ever higher status is not adaptive. This is because competing for status involves entry into zero-sum contests that can be lost as well as won. Accordingly, the pursuit of status is risky. Potential costs range from the psychologically uncomfortable (Ridgeway and Berger, 1986) to the physically lethal (Wrangham and Wilson, 2004). The upshot is that individuals must *pursue status judiciously*.

Consider, by analogy, the game of poker (Sklansky, 1994). Here, players compete to win a pot of money. Each player is dealt a private hand of cards, some of which, in certain combinations, are of higher value than others, leading to “good hands” versus “bad hands.” One player begins the round by placing a bet, and all players then respond in turn. Each player may either *raise* (i.e., increase the amount bet), *fold* (i.e., opt out of the round, losing the amount bet), or *check* (i.e., stay in the game, matching the amount bet). The round continues until only one player remains, who then wins the pot, or until two or more players remain, in which case the player with the best hand then wins the pot in a public showdown.

In poker, players play each round by considering their own cards, their opponents’ likely cards, and the relevant stakes. On this basis, they decide to raise or fold (checking being the intermediate option). Similarly, in social life, individuals navigate status hierarchies by considering their own abilities, their opponents’ likely abilities, and the costs and benefits of competition. On this basis, they decide whether or not to enter a contest, and whether or not to persist in it. In poker, players generally adopt the strategy of raising when their hands seem comparatively good, and folding when their hands seem comparatively poor. That is, they “know when to hold ‘em, know when to fold ‘em” (Schlitz, 1978). Similarly, in social life, people generally adopt the strategy of engaging in contests they expect to win and avoiding contests they expect to lose (Sloman and Price, 1987; Gilbert et al., 1995). That is, individuals, based on their best reckonings, either *escalate* (i.e., intensify) contests or *de-escalate* (i.e., defuse) contests. In humans, escalation requires more *assertive* behavior, whether in attack or defense; de-escalation, in contrast, requires more *acquiescent* behavior, whether in surrender or withdrawal. These alternative behavioral strategies have also been termed *hawk* and *dove* (Maynard Smith, 1982). The former offers higher reward at greater risk, the latter lower reward at lesser risk. Both can be adaptive, depending on the circumstances.

Hierometer theory proposes that higher self-regard prompts the adoption of more hawkish strategies, and lower self-regard the adoption of more dovish ones. As such, self-regard is part of a dedicated system that evolved to regulate behavior adaptively when navigating status hierarchies. To be adaptive, self-regard must predict the relative success of hawkish strategies when higher, and of dovish strategies when lower. Logically, this can only be the case if self-regard does indeed track some characteristic that predicts the relative success of those strategies. What could that characteristic be? *Social status* is a leading contender. Here, we define it as the respect, admiration, and importance that society at large confers upon an individual (Magee and Galinsky, 2008; Fiske, 2010; Huo et al., 2010). Higher-status individuals can afford to adopt more hawkish strategies. This is because, in order for them to have attained higher status in the first place, several factors would have had to operate in their favor. Such factors might have included a fund of pre-existing resources to draw on (Magee and Galinsky, 2008), or a superior ability to produce goods and services (Klein, 2010). Where present, such propitious factors would objectively increase the likelihood that adopting a hawkish strategy—that is, escalating contests through assertive behavior—would work better. In poker terms, higher-status individuals would hold a “good hand,” making it more adaptive for them to “raise.” However, where such factors are absent, as they often are among lower-status individuals, a dovish strategy—de-escalating contests through acquiescent behavior—would work better. In poker terms, lower-status individuals would hold a “poor hand,” making it more adaptive for them to “fold.”

Note here some key differences between hierometer theory and *dominance theory* (Barkow, 1975, 1980), another alternative to sociometer theory (e.g., Leary et al., 2001). Dominance theory, plausibly interpreted, states that self-esteem tracks, not levels of social acceptance or relational value, but instead levels of “dominance” or “prestige,” by which some social or psychological, rather than behavioral, construct is meant. For example, according to Barkow (1975), “[to] evaluate the self as *higher* than others is to maintain self-esteem (p. 554)” and “[…] approbation and respect permit the self to evaluate itself as being of *higher standing* than others, thereby maintaining self-esteem (p. 555–556, italics added).” Barkow (1980) proposes that people pursue various “prestige strategies” to maintain their standing, and draws analogies with social rank in other species. Accordingly, the term “status” might be reasonably substituted for “dominance” or “prestige” here, especially given the key role that attention and respect play in Barkow’s characterization (Anderson et al., 2001, 2006; Fiske, 2010). If so, then dominance theory, like hierometer theory, states that higher social status promotes higher self-esteem. However, insofar as dominance theory emphasizes the critical importance of social status for reproductive success, it can be interpreted as proposing that self-regard operates *homeostatically*, motivating individuals to attain and maintain sufficient levels of social status (Anderson et al., 2015), much as sociometer theory proposes happens with respect to levels of social inclusion (Baumeister and Leary, 1995). In contrast, hierometer theory proposes that self-regard operates *non-homeostatically*, regulating the behavior strategies that individuals adopt so that they match rather than modify their current status. Hence, dominance theory and hierometer theory make distinct predictions. The former predicts that lower self-regard, tracking lower status, should lead to increased behavioral assertiveness, as a form of adaptive *compensation*. The latter predicts that lower self-regard, tracking lower status, should lead to increased behavioral acquiescence, as a form of adaptive *consolidation*. Thus, hierometer theory, far from being a restatement of dominance theory, is an empirically testable alternative to it.

In brief, hierometer theory proposes that self-regard acts as a crucial psychological mediator: it bridges the gap between social status and assertive behavior by tracking the former and regulating the latter, thereby enabling individuals to navigate status hierarchies, as their status best allows. Fundamentally, then, hierometer theory is a theory of *winners* and *losers*.

### Hierometer Theory: Existing Evidence

Hierometer theory already enjoys some empirical support. Assorted findings are consistent with self-regard serving both its hypothesized indicative and imperative functions.

### Indicative Function: Tracking Social Status

Socioeconomic indicators (i.e., income) modestly predict trait self-esteem (Twenge and Campbell, 2002). In addition, state self-esteem increases or decreases respectively when students are notified of academic successes or failures (Crocker et al., 2002, 2003), when job candidates encounter a more or less smartly dressed competitor (Morse and Gergen, 1970), and when participants are assigned to the roles of supervisors or subordinates (Wojciszke and Struzynska-Kujalowicz, 2007, Study 2). Finally, from a neurological point of view, some brain circuits seem to be specialized to identifying one’s place in the status hierarchy (Zink et al., 2008).

### Imperative Function: Regulating Assertive Behavior

Trait self-esteem positively predicts self-reports of more assertive behavior (“interpersonal dominance”), both on standard questionnaires (Leary et al., 2001) and on behavioral vignettes (De Waal-Andrews, 2012, unpublished). In addition, when participants are alternately instructed to recall occasions on which they “felt secure in their self-worth” versus “felt like a failure”—thereby manipulating their state self-esteem—they recall more assertive behaviors and acquiescent behaviors respectively (De Waal-Andrews, 2012, unpublished).

Several studies also show that higher and lower self-regard are respectively associated with the adoption of more risk-seeking and risk-averse strategies. For example, individuals with higher self-esteem prefer risky gambles over certain gains on a monetary task (Josephs et al., 1992), at least when potentially regret-inducing feedback on foregone options is offered. Moreover, when telling a joke or completing a creativity test, they are more likely to “go for gold” than to “play it safe”—an effect amplified under conditions of psychological threat (Landau and Greenberg, 2006). Individuals with high self-esteem also make riskier personal decisions on interpersonal vignettes even controlling for trait anxiety, with the effect being partly mediated by success expectancies and failure-related feelings (Wray and Stone, 2005).

### The Sociometer and the Hierometer: Toward Conceptual Coordination

How do sociometer theory and hierometer theory relate to one another? Are they antagonistic or complementary? The answer, it turns out, hangs on how one *interprets* sociometer theory, and on *which formulation* of it one emphasizes, for one ends up dealing with theoretical constructs that are broader or narrower in scope, and defined with greater or lesser precision. Our summary of sociometer theory above blends (intentionally) *two versions* of the theory, original and revised.

The original version of sociometer theory (Leary and Downs, 1995; Leary et al., 1995) emphasizes how self-esteem tracks social acceptance, by which is implied some sort of community belongingness, or *social inclusion*. For example, Leary (2004, p. 374), recounting the original version, states that “only those who have established *mutually supportive* relationships with people can count on others’ *assistance* in terms of food *sharing*, physical protection, and *care* when ill, injured, or old. An individual who does not maintain a minimal level of social acceptance is at a decided disadvantage compared to one who is *warmly* accepted […] humans […] possess a strong and pervasive need for acceptance and *belongingness* […] [italics added].”

In contrast, the revised version (Leary and Baumeister, 2000) emphasizes how self-esteem tracks *relational value*, defined as the degree to which other people regard their relationship with the individual as *important or valuable overall*, for whatever reason. For example, Leary (2005, p. 86) states that “*Many* kinds of events can lower people’s self-esteem—failure, rejection, embarrassing situations, negative evaluations, being outperformed by others, and so on—but, from the standpoint of sociometer theory, these *all* involve events that potentially lower people’s relational value in the eyes of others [italics added]” (see also Kirkpatrick and Ellis, 2001; Leary et al., 2001, p. 907).

Accordingly, the revised version of sociometer theory is pitched at a broader level of analysis than the original version is. Relational value, as a construct, is broad enough to cover *any* reason for self-esteem’s rising and falling that involves social interaction. However, social inclusion, as a construct if understood as community belongingness, is narrower, and *excludes* many such reasons. In particular, one can enjoy high status, by being respected or admired for being competent, without enjoying high inclusion, by being liked or loved for being warm—and vice versa (Cuddy et al., 2008). However, the evidence suggests that status, so conceived, can affect self-esteem independently of inclusion, so conceived (e.g., Leary et al., 2001: Study 1; Koch and Shepperd, 2008). Consequently, the original version of sociometer theory cannot account for such findings, only the revised version. More generally, the original version characterizes self-esteem primarily in terms of the super-dimension of *communion*, which comprises such factors as inclusion, warmth, and affiliativeness, rather than in terms of the (orthogonal) super-dimension of *agency*, which comprises such factors as status, competence, and assertiveness (Foa, 1961; Wiggins, 1979; McCrae and Costa, 1989; Moskowitz, 1994; Cuddy et al., 2008; Huo et al., 2010). This is problematic, because self-esteem may actually be *more* agency-based. For example, Wojciszke et al. (2011) found that self-ascribed agentic traits (e.g., “clever”) predicted self-esteem better than self-ascribed communal traits (e.g., “good-natured”; though see Gebauer et al., 2013, for some moderators). In addition, Zeigler-Hill (2010) reported that eight standard measures of self-regard were at least as strongly linked to agentic traits as they were to communal ones.

Hence, it is important to be theoretically precise about self-esteem’s agentic role. The original version of sociometer theory neglects it by emphasizing community belongingness. The revised version partly rectifies this neglect by allowing for the possibility of many types of relational value. However, the revised theory still does not specify the details of self-esteem’s agentic role, nor does it differentiate self-esteem’s agentic role from its communal role. Hierometer theory breaks new theoretical ground by specifying precisely how (social) status, (psychological) self-esteem, and (behavioral) assertiveness might interact as part of an evolutionarily adaptive system. Moreover, with this agentic role specified, the communal role played by self-esteem comes into sharper focus. In particular, the original version of sociometer theory can be plausibly interpreted as specifying precisely how (social) inclusion, (psychological) self-esteem, and (behavioral) affiliativeness might interact as part of evolutionarily adaptive system. Thereafter, it becomes possible to empirically test whether and to what extent these agentic and communal roles are supported or refuted by patterns of empirically observed correlations.

A further virtue of hierometer theory’s specificity is that, in clearly distinguishing between different levels of analysis, including in the original version of sociometer theory, it encourages the operationalization of constructs at corresponding levels, a finesse that some prior research has not observed. For example, Koch and Shepperd (2008), in testing how well-acceptance (i.e., inclusion) and competence independently predicted self-esteem, although they tested a communal construct against an agentic one, nonetheless confounded social and psychological levels of analysis, respectively. Arguably, they should have tested how inclusion versus status, or competence versus warmth, predicted self-esteem. In addition, Anthony et al. (2007a), when testing how well different types of traits—namely, “communal qualities” versus “social commodities”—independently predicted self-esteem, listed “popular” and “social status” as examples of the latter. However, popularity and status are not only psychological in nature, but also social.

### Hierometer Theory: Encompassing Narcissism Too

We have so far alternated between using the term “self-regard,” mainly in respect of hierometer theory, and the term “self-esteem,” mainly in respect of sociometer theory. And intentionally so: for self-regard is a broader construct than self-esteem, and hierometer theory explicitly allows for the possibility that other types of self-regard might serve its prescribed indicative and imperative functions. We have one particular candidate in mind: *narcissism*.

Narcissism, for our purposes, is a normally distributed trait, and may be regarded as the continuous subclinical counterpart of the categorical personality disorder (Baumeister et al., 2003; Kernis, 2003; Campbell and Foster, 2007; Miller et al., 2014). Its antecedents, correlations, and consequences have been extensively researched (Paulhus et al., 2004; Rhodewalt and Peterson, 2009; Campbell and Miller, 2011), often in conjunction with self-esteem (Sedikides et al., 2004; Lee et al., 2013, Study 1). As most commonly operationalized (but see Pincus et al., 2009, for one of several alternatives), narcissism entails an interest in leadership and authority, a propensity for grandiosity and exhibitionism, and a sense of entitlement combined with a manipulative streak (Raskin and Hall, 1981; Ackerman et al., 2011). In romantic relationships, moreover, narcissists prefer being admired over being loved, pursue short-term rather than long-term relationships, and evaluate mates based on external characteristics rather than inner qualities (Campbell, 1999; Brunell and Campbell, 2011; Holtzman and Strube, 2011). Accordingly, narcissism has been hypothesized to involve a surfeit of agency alongside a deficit in communion (Campbell et al., 2002; Sedikides et al., 2002; Morf et al., 2011).

If this characterization is correct, then one might expect narcissism to be particularly likely to track social status and regulate assertive behavior, as hierometer theory specifies. Conversely, one might expect narcissism to be less likely to track social inclusion and regulate affiliative behavior, as sociometer theory specifies. Thus, hierometer theory, by encompassing narcissism too, opens up fruitful avenues for empirical investigation (cf. Back et al., 2009) and adds to the literature exploring narcissism’s evolutionary origins (Holtzman and Strube, 2011).

It should be noted that attempts have been made to account for narcissism in terms of sociometer theory. In particular, narcissism in this connection has been described as a *dysfunction*, where those who exhibit it believe that “others regard them more favorably and accept them more enthusiastically than is, in fact, the case” (Leary and Downs, 1995, p. 138). In other words, levels of self-regard in narcissists are no longer an accurate guide to their objective levels of social inclusion or relational value. But if so, then sociometer theory does not so much account for narcissism as undergo qualification in the light of its existence; for in narcissists, self-regard does *not* function as sociometer theory prescribes. In contrast, hierometer theory offers a positive and constructive account of the function of narcissism, which can be put to the empirical test.

To be clear, we regard self-esteem as the prototypical form of self-regard, and thus the primary construct to consider in evaluating functional theories. Nonetheless, narcissism is still perhaps the best known subtype of self-regard, and its concurrent consideration—especially given the affinity between the agentic emphasis of hierometer theory and the agentic roots of narcissism—is well-justified, and potentially enlightening.

### Goals and Features of the Current Research

Here, we report a systematic program of correlational research with several interlocking goals and features.

First, the research program is designed to test hierometer theory, by examining whether and to what extent its core constructs—status, self-regard, and assertiveness—interrelate as predicted. Second, it is designed to test sociometer theory in an exactly parallel manner. Do its core constructs—inclusion, self-regard, and affiliativeness—also interrelate as predicted? Third, the program operationalizes relevant constructs at various levels of analysis, by distinguishing among two types of *social relations* (i.e., status, inclusion), *psychological self-perception* (i.e., self-esteem, narcissism), and *behavioral strategy* (i.e., assertiveness, affiliativeness). Fourth, the program features a comprehensive analytic approach, to meet the goals above. To begin with, it looks at simple and partial correlations between (a) social relations and self-regard, and (b) self-regard and behavioral strategy. This permits separate tests of whether the data support or refute the indicative and imperative functions hypothesized by hierometer theory and sociometer theory. Fifth, the program proceeds to test—for the first time we believe—whether and to what extent self-regard *mediates* the link between social relations and interpersonal behavior, as hierometer theory and sociometer theory respectively predict. Sixth, in testing the above, the program investigates self-regard, not only in its standard form as self-esteem, but also in a grandiose form as narcissism. Finally, the program proceeds to investigate whether and to what extent any empirically confirmed links to self-esteem continue to obtain after statistically controlling for clinical variables known to overlap with self-esteem—in particular, depression and anxiety—which have themselves, in the context of evolutionary theory, been hypothesized to serve similar indicative and imperative functions (Price et al., 1994; Sloman, 2008).

It should be understood that this program of research represents the first step in a process of validating hierometer theory in the comparative context of sociometer theory. It allows for the possibility that patterns could emerge that are at odds with one or both theories, and enables tests of whether the data fit one theory better than another or both equally. Note that a complementary program of experimental research is also underway to test the hypothesized causal pathways between the social, psychological, and behavioral variables more definitively. For example, Mahadevan et al. (2015) found that expectations of higher and lower future social status *or* inclusion—both manipulated via bogus test feedback—independently led to higher and lower self-esteem, thereby validating the indicative functions of self-esteem specified by both hierometer theory and sociometer theory.

### Hypotheses

In sum, in regards to hierometer theory, we hypothesized that status would correlate positively with self-esteem as well as narcissism; that self-esteem and narcissism would each correlate positively with assertiveness; and that self-esteem and narcissism would each mediate the link between status and assertiveness. In regards to sociometer theory, we hypothesized that inclusion would correlate positively with self-esteem. Evidence for the link between self-esteem and affiliativeness is mixed. However, as low self-esteem is theorized to prompt greater affiliativeness in order to repair levels of social inclusion, we tentatively hypothesized that self-esteem would correlate negatively with affiliativeness. Furthermore, we hypothesized that self-esteem would mediate the link between inclusion and affiliativeness.

## Materials and Methods

### Overview

We conducted two studies. Each featured shared and unique elements. The shared elements were designed repeatedly to test our focal hypotheses concerning hierometer theory and sociometer theory. To this end, both studies assessed social relations (status and inclusion), psychological self-perception (self-esteem and narcissism), and behavioral strategy (assertiveness and affiliativeness). The unique elements of the studies were designed to rule out plausible alternative hypotheses and to advance theoretical understanding. To this end, Study 1 also assessed depression, and Study 2 additionally also anxiety. Regarding the shared elements of both studies, we present their associated analyses in parallel. This makes for greater brevity and permits the replicability of findings to be scrutinized. Regarding the unique elements of both studies, we present their associated analyses in sequence. This makes for a systematic analytic progression.

### Platform, Procedure, Precautions, and Participants

All studies were run online. They were created and administered using the designated university internet survey software. Participants were recruited via *CrowdFlower*^TM^, a leading crowdsourcing site, and took part for a nominal fee, on condition they were aged 18 years or above and were fluent in English. Before taking part, participants read some introductory information and indicated their consent; afterward, they read a debriefing statement. The studies were approved by the Research Ethics Committee of the University of Southampton, UK.

Crowdsourcing typically yields high-quality data, both rapidly and cheaply, from diverse participants (Buhrmester et al., 2011; Germine et al., 2012). Nonetheless, it is prudent to exclude participants whose data are, for any of several reasons, suspect. Here, we excluded participants if: (a) their IP address appeared more than once in the dataset (suggesting multiple completions); (b) they completed the survey in less than half the median survey time (suggesting an absence of reflection); (c) they completed all items identically on any questionnaire featuring both forward-scored and reverse-scored items (suggesting mindless button-clicking); (d) their self-reported English-proficiency was poor or very poor; (e) they were younger than 18; and (f) they omitted to answer more than 5% of questionnaire items.

**Table 1** shows sample demographics before and after screening, and the percentage excluded for each reason. As some participants were excluded for multiple reasons, the sum of the individual percentages excluded exceeds the total percentage. Over 85% of the original participants were retained. Of these, about two-thirds were female, nine-10ths resident in the USA, and the majority aged between 20 and 40.^2^ We report analyses conducted on screened data, although analyses conducted on unscreened data yielded inferentially equivalent results.

**Table 1:** Participant profile.

Variable	Study 1	Study 2
**Total sample size**	**712**	**789**
Duplicate IP addresses	2.7%	2.9%
Age <18 years	0.8%	1.0%
Poor reported English proficiency	0.7%	1.4%
Overly rapid completion (< half median completion time)	3.7%	5.8%
>5% blank	4.8%	4.2%
Stereotyped responses	2.5%	3.7%
**Total excluded**	**12.1%**	**13.8%**

**Screened sample size**	**626**	**680**
Gender (male)	37.5%	39.1%
Mean age (in years)	34.5	32.3
SD age (in years)	12.9	12.8
U.S. residence	91.2%	86.5%

### Measures

#### Social Relations

We assessed status and inclusion, respectively, with structurally validated 8- and 9-item scales. These scales were originally developed by Huo et al. (2010) and enhanced by Mahadevan et al. (2015) with additional items that gave a broader conceptual coverage and achieved a superior factor solution. Each item began with the stem “Most of the time I feel that people*…*” and ended with a different sentence completion (e.g., status: “…see me as an important person”; inclusion: “…like me as a person”). Both scales featured a five-point response format (1 *= strongly disagree*, 5 = *strongly agree*).

#### Psychological Self-Regard

We assessed self-esteem with the 10-item *Rosenberg Self-Esteem Scale* (*RSES*; Rosenberg, 1965). We assessed narcissism with one of two versions of the *Narcissistic Personality Inventory*: the original 40-item (multifactorial) version (*NPI-40*: Raskin and Hall, 1981; Studies 1 and 3) or the abbreviated (unifactorial) 16-item version (*NPI-16*; Ames et al., 2006; Study 2).^3^ Both the RSES and the NPI are leading measures of self-regard (Byrne, 1996; Twenge et al., 2008). The RSES featured a five-point response format (1 = *strongly disagree*, 5 = *strongly agree*), and the NPI a six-point sliding scale, located between bipolar options (cf. Lee et al., 2013). Sample items: “I feel that I have a number of good qualities” (RSES); “I know that I am good because everybody keeps telling me so” versus “When people compliment me I sometimes get embarrassed” (NPI).

#### Behavioral Strategy

We assessed assertiveness and affiliativeness using the 48-item *Social Behavior Inventory* (*SBI*; Moskowitz, 1994). The SBI consists of four 12-item subscales that respectively assess behaviors originally labeled *dominant, submissive, agreeable*, and *quarrelsome* (although we prefer the labels *assertive*, *acquiescent*, *affiliative*, and *alienating*, which emphasize interpersonal action over individual disposition, and avoid conceptual confounds [e.g., *dominant* implies a social rank]). The SBI featured a six-point response format (1 = *very unlike me*, 6 = *very like me*). Sample items: “I speak in a clear, firm voice” (dominant); “I do not express disagreement” (submissive); “I compliment or praise other people” (agreeable); and “I criticize others” (quarrelsome). To derive an overall assertiveness index, we subtracted participants’ mean score on the submissive subscale from their mean score on the dominant subscale; to derive an overall affiliativeness index, we subtracted participants’ mean score on the quarrelsome subscale from their mean score on the agreeable subscale.

#### Depression

We assessed depression in two ways: with the 21-item *Beck Depression Inventory-II* (*BDI-II*; Beck et al., 1996) and with the 20-item *Centre for Epidemiological Studies Depression Scale* (*CES-D*; Radloff, 1977). The BDI-II lists different symptoms, followed by topic-specific response options each time. Sample item: “Sadness”: (1 = *I do not feel sad*; 2 = *I feel sad some of the time*; 3 = *I am sad all the time*; 4 *= I am so sad or unhappy I can’t stand it*). The CES-D lists different statements about symptoms, followed by standard response options. Sample item: I was bothered by things that usually don’t bother me” (1 = *Never or hardly ever;* 2 = *Occasionally, now and then;* 3 *= A good deal of the time;* 4 = *Mostly or all of the time*). Given the high correlation between the BDI-II and the CES-D, *r*(624) = 0.85, *p* < 0.0001, we created a single composite measure, by standardizing scores on each and averaging the result.

#### Anxiety

We assessed anxiety in two ways: with the 21-item *Beck Anxiety Inventory* (*BAI*; Beck et al., 1988) and with the 20-item trait version of the *State Trait Anxiety Inventory* (*STAI*; Spielberger et al., 1983). Both measures cover a range of anxiety symptoms (e.g., nervousness, light-headedness, trembling), are suitable for clinical and non-clinical samples, and feature four-point response formats. The BAI lists the names of different anxiety symptoms, and has respondents indicate their severity. Sample item: “Numbness or tingling” (1 = *NOT AT ALL: It didn’t bother me in the slightest*; 2 = *MILDLY: It didn’t bother me much*; 3 = *MODERATELY: It wasn’t pleasant at times*; and 4 = *SEVERLY: It bothered me a lot*). The STAI lists different statements about dispositional anxiety, and has respondents indicate their level of agreement. All statements began with the sentence stem “In general....” Sample item: “…I worry over possible misfortunes” (1 = *Not at all*; 2 = *A little*; 3 = *Somewhat*; and 4 = *Very much so)*. Again, given the high correlation between the BAI and the STAI, *r*(678) = 0.64, *p* < 0.0001, we created a single composite measure, by standardizing scores on each and averaging the result.

## Results and Discussion

### Descriptive Statistics

**Table 2** summarizes the key aspects of all measures (means, standard deviations, internal consistencies, deviations from midpoint). In no case did any measure fall short of conventional psychometric desiderata. Moreover, no variable evinced a distribution that was atypical for everyday populations (e.g., levels of self-esteem were significantly higher than the midpoint, and levels of narcissism were significantly lower than the midpoint).

**Table 2:** Means, standard deviations, and reliabilities of main variables.

		Study 1			Study 2		
Measures	Scale	*M*	*SD*	α	*M*	*SD*	α
Status	1 – 5	3.21 ↑	0.75	0.91	3.33 ↑	0.75	0.91
Inclusion	1 – 5	3.68 ↑	0.71	0.93	3.75 ↑	0.67	0.92
Self-esteem	1 – 5	3.59 ↑	0.84	0.92	3.61 ↑	0.79	0.91
Narcissism	1 – 6	3.07 ↓	0.71	0.92	3.03 ↓	0.71	0.81
Assertiveness	1 – 6	3.80 ↑	0.81	0.93	3.91 ↑	0.76	0.92
Affiliativeness	1 – 6	4.48 ↑	0.61	0.89	4.51 ↑	0.59	0.88
Depression (BDI-II)	1 – 4	1.59 ↓	0.54	0.94	–	–	–
Depression (CES-D)	1 – 4	1.88 ↓	0.60	0.93	–	–	–
Anxiety (BAI)	1 – 4	–	–	–	1.81 ↓	0.67	0.95
Anxiety (STAI)	1 – 4	–	–	–	2.15 ↓	0.66	0.95

### Indicative Function: Social Relations and Psychological Self-Regard

Both hierometer theory and sociometer theory predict that levels of psychological self-regard will track levels of social relations (i.e., serve an *indicative* function). Hierometer theory specifically predicts that higher status will lead to higher self-regard, either as self-esteem or narcissism. Sociometer theory, in its original version, specifically predicts that higher inclusion will lead to higher self-regard in the form of self-esteem, though it makes no prediction concerning narcissism (but see Leary and Downs, 1995). However, to the extent that narcissism, unlike regular self-esteem, entails an agentic surfeit and a communal deficit, narcissism should disproportionately track status over inclusion, whereas self-esteem should track both more equitably.

The upper panel of **Table 3** lists all correlations between the two social relations variables (i.e., status and inclusion) and the two self-regard variables (i.e., self-esteem and narcissism). These appear in two forms: raw (first row) and partialed (second row). The partial correlations between any pair of variables (e.g., status and narcissism) controlled for overlapping variance attributable to the other pair (e.g., inclusion and self-esteem).

**Table 3:** Raw and partial correlations between social relations, self-regard, and interpersonal behavior.

	Study 1		Study 2	
	Self-esteem	Narcissism	Self-esteem	Narcissism
**Raw correlations**				
Status	0.63^∗∗^	0.48^∗∗^	0.61^∗∗^	0.35^∗∗^
Inclusion	0.55^∗∗^	0.31^∗∗^	0.55^∗∗^	0.17^∗∗^
**Partial correlations**				
Status	0.35^∗∗^	0.28^∗∗^	0.35^∗∗^	0.25^∗∗^
Inclusion	0.25^∗∗^	-0.05	0.29^∗∗^	-0.11^∗^
	
	**Assertiveness**	**Affiliativeness**	**Assertiveness**	**Affiliativeness**
	
**Raw correlations**				
Self-esteem	0.48^∗∗^	0.25^∗∗^	0.39^∗∗^	0.21^∗∗^
Narcissism	0.62^∗∗^	-0.17^∗∗^	0.50^∗∗^	-0.34^∗∗^
**Partial correlations**				
Self-esteem	0.29^∗∗^	0.36^∗∗^	0.27^∗∗^	0.32^∗∗^
Narcissism	0.50^∗∗^	-0.26^∗∗^	0.40^∗∗^	-0.40^∗∗^

*^∗^p < 0.01; ^∗∗^p < 0.001.*

Several patterns deserve note. First, all raw coefficients were significantly positive. At first glance, then, the data are consistent with both forms of self-regard simply tracking both forms of social relations. The higher participants’ status or inclusion, the higher their self-esteem or narcissism was too. Second, the same raw coefficients were, within each study, always higher for self-esteem than for narcissism. This suggests that self-esteem is, overall, a more sensitive tracker of both status and inclusion. Third, the raw coefficients were, within each study—and broken down by self-esteem and narcissism individually—always at least somewhat higher for status than for inclusion. This suggests that self-regard tracks status at least as sensitively as inclusion. Finally, the difference in raw coefficient size for correlations with status and inclusion was greater for narcissism than it was for self-esteem. This suggests that, compared to self-esteem, narcissism is a relatively more sensitive tracker of status than inclusion (while being an absolutely less sensitive tracker of both).

Nonetheless, status and inclusion correlated positively in both studies—respective *rs* = 0.64 and 0.63, *p*s < 0.0001—as did self-esteem and narcissism—respective *rs* = 0.43 and 0.30, *p*s < 0.0001. Accordingly, we examined the partialed coefficients to gage the crucial links between the statistically “purified” variables.

Although predictably lower in magnitude, all partial coefficients nonetheless remained positive and significant—with one key and consistent exception: narcissism now correlated negatively with inclusion, and significantly so in one out of two studies. This suggests that, if anything, narcissism tracks decrements in inclusion by increasing, not by decreasing (like self-esteem). This novel finding underscores the value of assessing self-regard in two different ways, and raises the possibility that any functional link between self-regard and inclusion may not be monolithic and unidirectional. The overall picture is that, whereas self-esteem increases with better social relations generally, narcissism increases with one form (status) but decreases with the other form (inclusion). Otherwise, the patterns obtained with partialed coefficients mirrored those obtained with raw coefficients, suggesting identical conclusions.

In all, the patterns obtained were consistent with self-regard serving the indicative functions specified by hierometer theory (i.e., for self-esteem and narcissism) and by sociometer theory (i.e., for self-esteem). Importantly, however, both forms of self-regard independently covaried with status at least as much as with inclusion; accordingly, self-regard, if it tracks social relations, seems not only to track community belongingness, but also and no less sensitively, achievement-related standing. Moreover, the inverse link between narcissism and inclusion complicates the empirical picture: it suggests a new and unanticipated type of indicative function, not easily accounted for in terms of the original version of sociometer theory. The findings also support the characterization of narcissism in terms of an agentic surfeit and communal deficit.

### Imperative Function: Psychological Self-Regard and Behavioral Strategy

Both hierometer theory and sociometer theory predict that levels of psychological self-regard will regulate behavioral strategies (i.e., serve an *imperative* function). The lower panel of **Table 3** lists, for both studies, the correlations between the two self-regard variables (i.e., self-esteem and narcissism) and the two behavioral strategy variables (i.e., assertiveness and affiliativeness). Again, these appear in two forms: raw (third row) and partialed (fourth row). The partial correlations between any pair of variables (e.g., narcissism and assertiveness) controlled for overlapping variance attributable to the other pair (e.g., self-esteem and affiliativeness). Here, assertiveness and affiliativeness emerged as largely orthogonal, respective *rs* = -0.05 and -0.07, *p*s > 0.05. Nonetheless, for the sake of consistency, we applied the same partialing procedure.

All correlation coefficients were significant. For both raw and partialed correlations, the higher participants’ self-esteem, the higher was their assertiveness and affiliativeness; but the higher their narcissism, the higher was their assertiveness and the lower their affiliativeness. Thus, the data were consistent with both forms of self-regard regulating both forms of behavioral strategy, albeit in different ways. They suggest that whereas self-esteem regulates assertiveness and affiliativeness similarly (by making each rise as it rises), narcissism regulates them differently (by making assertiveness rise, but affiliativeness fall, as it rises).

As regards other patterns, the data did not strongly support either form of self-regard being a more potent regulator of behavioral strategies overall, or either form of behavioral strategy being more potently regulated by self-regard overall. The narcissism-assertiveness coefficients were always (absolutely) the highest; the others were closer in magnitude to one another, especially in their partialed form. Among the partialed coefficients, a cross-over trend could be discerned, such that self-esteem covaried (slightly) more strongly with affiliativeness than assertiveness, whereas narcissism covaried (much) more strongly with assertiveness than affiliativeness. As before, narcissism seemed to have been characterized by agentic surfeit and a communal deficit.

How then do these findings bear on the validity of the hierometer theory and sociometer theory? As regards hierometer theory, all the findings are fully consistent with it. As both forms of self-regard increased, assertiveness increased; and as both forms of self-regard decreased, assertiveness decreased. These findings suggest a form of adaptive consolidation, with higher or lower levels of self-regard inducing individuals to adopt, respectively, a more assertive or acquiescent behavioral strategy, in keeping with the competitive capacities liable to be conferred by their higher or lower status, and tracked by self-regard. In contrast, dominance theory (Barkow, 1975, 1980) arguably implies the reverse: lower levels of self-regard should have prompted adaptive compensation, inducing individuals to adopt a more assertive behavioral strategy to augment their diminished status. Accordingly, our findings support hierometer theory over dominance theory.

As regards the original version of sociometer theory, the same logic of adaptive compensation versus adaptive consolidation applies, but concerning inclusion and affiliativeness rather than status and assertiveness. Individuals whose self-esteem is lower, due to their lower levels of inclusion, should be more inclined to adopt affiliative behavioral strategies designed to rectify their lower levels of inclusion, whereas those whose self-esteem is higher, due to their higher levels of inclusion, can afford to relax the imperative to affiliate. However, our findings pointed toward the opposite conclusion: levels of self-esteem covaried *positively*, not negatively, with affiliativeness. This is consistent with a consolidatory rather than a compensatory dynamic. Social exclusion seems to have an alienating impact: it reduces affiliativeness, which is arguably less adaptive in the absence of benevolent and beneficent reciprocators. Such a dynamic would fit with some empirical findings (e.g., Twenge et al., 2007), although not with others (e.g., Maner et al., 2007). Persistent failure to achieve inclusion—which our chronically oriented measures were liable to assess—does seem to promote hostility (DeWall and Richman, 2011).

However, given that narcissism covaried negatively with affiliativeness, it fitted the bill, sociometer-wise, where self-esteem did not. In particular, as narcissism decreased, participants adopted a more affiliative behavioral strategy. This suggests that narcissism might operate in the imperative manner prescribed by the original version of sociometer theory. However, as noted above, narcissism does not operate in the prescribed indicative manner: it also covaried negatively with social inclusion. Hence, narcissism cannot entirely operate as the original version of sociometer theory specifies self-esteem should.

It might be objected that sociometer theory only properly applies to state self-esteem, not to trait self-esteem; and indeed, the bulk of research cited in support of sociometer references state self-esteem (e.g., Leary, 2005). However, associations with trait self-esteem have previously been marshaled in support of sociometer in respect of its prescribed indicative functions (e.g., Leary et al., 1995, 2001; Anthony et al., 2007a; Denissen et al., 2008). This makes it rhetorically difficult to object to their bearing on its prescribed imperative functions also. A case would have to be made for why—if sociometer theory is correct about social inclusion being so essential for survival— individuals who are chronically low in self-esteem, due to chronic social exclusion, should not strive for re-inclusion by adopting a chronically affiliative behavioral strategy.

In all, the patterns obtained were consistent with self-regard serving the imperative functions specified by hierometer theory (for self-esteem and narcissism) but not those specified by sociometer theory (for self-esteem).

### Mediational Analyses I: Self-Esteem and Narcissism

By considering above the separate links between (a) social relations and psychological self-regard, and (b) psychological self-regard and behavioral strategy, we tested, in each case, whether the data were either consistent or inconsistent with self-regard serving the different indicative and imperative functions specified by hierometer theory and (the original version) of sociometer theory. However, both theories additionally specify that the indicative and imperative functions are *coordinated*: social relations should affect self-regard, which should in turn affect behavioral strategy. If so, then there should be a link between social relations and behavioral strategy, which is mediated by self-regard.

**Table 4** lists, for both studies, all correlations between the two social relations variables (i.e., status and inclusion) and the two behavioral strategy variables (i.e., assertiveness and affiliativeness). These appear in two forms: raw (top row) and doubly partialed (bottom row).

**Table 4:** Raw and partial correlation between social relations and interpersonal behavior.

	Study 1		Study 2	
	Assertiveness	Affiliativeness	Assertiveness	Affiliativeness
**Raw correlations**				
Status	0.42^∗∗^	0.22^∗∗^	0.40^∗∗^	0.13^∗∗^
Inclusion	0.35^∗∗^	0.35^∗∗^	0.31^∗∗^	0.31^∗∗^
**Partial correlations**				
Status	0.28^∗∗^	0.06	0.26^∗∗^	-0.04
Inclusion	0.16^∗∗^	0.30^∗∗^	0.14^∗∗^	0.32^∗∗^

*^∗∗^p < 0.001.*

The patterns—especially the raw coefficients—show that, generally speaking, higher status and inclusion covaried positively with assertiveness and affiliativeness. Thus, better social relations generally prompted higher levels of both behavior strategies. Beyond this—as the partialed coefficients best attest—status covaried relatively more strongly with assertiveness, and inclusion relatively more strongly with affiliativeness.

**FIGURE 2:**
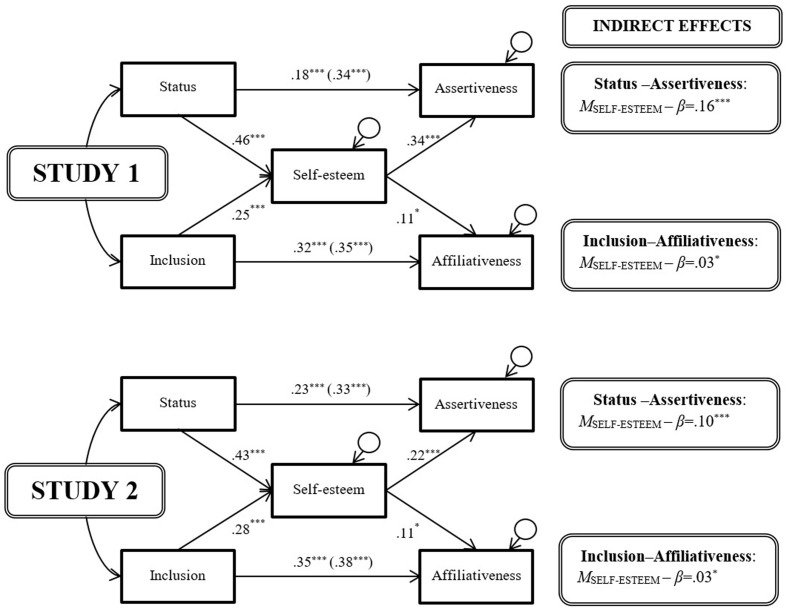
**The mediating effect of self-esteem.** ^†^*p* < 0.10; ^∗^*p* < 0.05; ^∗∗^*p* < 0.01; ^∗∗∗^*p* < 0.001.

**FIGURE 3:**
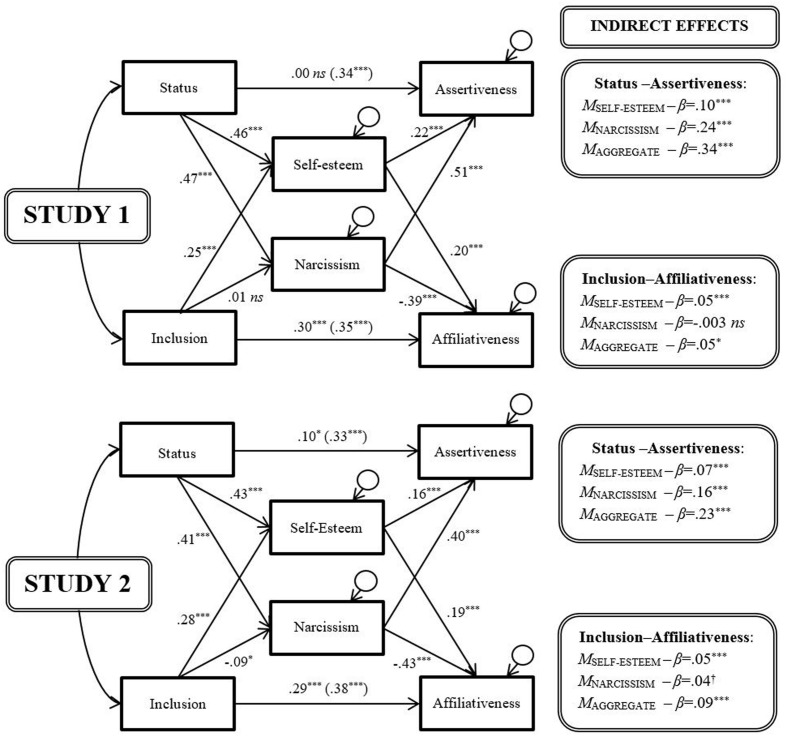
**The mediating effects of self-esteem and narcissism.** ^†^*p* < 0.10; ^∗^*p* < 0.05; ^∗∗^*p* < 0.01; ^∗∗∗^*p* < 0.001.

To test whether self-regard mediated these links, we constructed, for each study, a structural equation model (Kline, 2005). In both sets, we estimated all directional paths using bias-corrected bootstraps on standardized scores (Efron, 1987). As all models were fully saturated, and had zero degrees of freedom, no goodness of fit indices applied (Kline, 2005, p. 133). In the first set of models (**Figure 2**), we entered status and inclusion as a pair of exogenous predictor variables, self-esteem as an endogenous mediator variable, and assertiveness and affiliativeness as a pair of endogenous outcome variables. We permitted status and inclusion to covary, and we did the same for assertiveness and affiliativeness. In the second set of models (**Figure 3**), we added narcissism as a second endogenous mediator variable, and additionally permitted self-esteem and narcissism to covary.^4^^,^^5^

Across both studies, the findings from the first set of models (**Figure 2**) were remarkably consistent. Overall, self-esteem significantly and substantially mediated the link between social relations and behavioral strategy. Specifically, the indirect paths between status and assertiveness via self-esteem always attained significance, although the direct paths remained significant too. Likewise, the indirect paths between inclusion and affiliativeness via self-esteem always attained significance, although the direct paths remained significant too. Furthermore, all paths, direct and indirect, were positive in sign, suggesting a consolidatory dynamic, with higher status prompting greater assertiveness by raising self-esteem, and higher inclusion prompting greater affiliativeness in like manner. Finally, the magnitude of the mediation was more marked for the agentic variables invoked by hierometer theory than for the communal variables invoked by (the original version of) sociometer theory. In particular, the path from self-esteem to affiliative behavior was notably weak.

Across both studies, the findings from the second set of models (**Figure 3**) were also highly consistent. Overall, self-regard—represented by the combination of self-esteem and narcissism—again significantly and substantially mediated the link between social relations and behavioral strategy. However, the pattern of mediation differed markedly for the hierometer-relevant agentic variables and the sociometer-relevant communal variables. This time, the direct path between status and assertiveness dropped to zero in one case, showing that the self-regard “combo” completely mediated the link between status and assertiveness. (The incomplete mediation observed in Study 2 may have been due to its featuring, not the full 40-item version of the NPI, but rather the abbreviated 16-item version, which sacrificed conceptual coverage for administrative brevity.) Thus, the addition of narcissism to the models, which lowered direct effects from around 0.20 to 0, substantially augmented the mediation of the status-assertiveness link—again underscoring the value of assessing self-regard in two different ways. In contrast, the addition of narcissism to the model hardly affected the mediation of the inclusion-affiliativeness link: as before, direct effects of around 0.30 emerged. This was primarily because the paths from inclusion to narcissism were near-zero, despite the emergence of strong negative paths between narcissism and affiliativeness, and the positive paths from self-esteem to affiliativeness rising slightly.

This first set of mediational findings fully supports hierometer theory. The data are consistent with self-regard—especially when jointly operationalized as self-esteem and narcissism—acting as a psychological mediator that bridges the gap between status and assertiveness. In particular, they are consistent with self-regard tracking status (by rising or falling in tandem with it) and regulating assertiveness (by making it rise or fall in tandem) to help ensure that individuals navigate adaptively status hierarchies by judiciously engaging in zero-sum contests (i.e., as their status dictates).

Second, the mediational findings only offer at best partial support for the original version of sociometer theory. In the aggregate, the data arguably do not support it. Certainly, our data suggest that self-esteem tracks inclusion, by rising (or falling) in tandem with it. However, they also suggest that, at the same time, self-esteem makes affiliativeness rise (or fall) in tandem with it too. Sociometer theory would arguably predict the opposite, namely, that lower self-esteem should prompt greater affiliativeness, to help restore the fractured bonds of community belongingness, regarded as so fundamental to survival. Yet narcissism did vary inversely with affiliativeness, consistent with one variant form of self-regard regulating behavior in the required direction. At the same time, narcissism hardly covaried with inclusion, thereby ruling it out as a mediator to bridge the sociometer-specified gap between inclusion and affiliativeness.

### Mediational Analyses II: Self-Esteem, Depression, and Anxiety

Self-esteem is known to covary inversely with most negative emotions, in particular, with anxiety and depression (Baumeister et al., 2003; Sedikides et al., 2004). Furthermore, some theories (Price et al., 1994; Sloman, 2008) state that depression and anxiety evolved to serve indicative and imperative functions similar to those hypothesized by sociometer and hierometer theory to be served by self-esteem. In particular, depression *per se* has been hypothesized to activate a harm-minimizing “yielding subroutine” (Price and Sloman, 1987) that facilitates the emergence of stable “pecking orders” across species—rather like hierometer theory proposing that low-esteem curtails assertiveness to optimize competitive performance within social hierarchies. In addition, social anxiety *per se* has been hypothesized to keep people appropriately mindful of what is socially acceptable lest they fall prey to public sanction—similar to sociometer theory proposing that low self-esteem signals to people their levels of social inclusion or relational value are running dangerously low (Gilbert, 2001). Although self-esteem might conceivably operate in concert with anxiety and depression, both hierometer and sociometer theory would be strongly validated if findings emerged that were consistent with self-esteem playing a mediational role independently of anxiety or depression.

**FIGURE 4:**
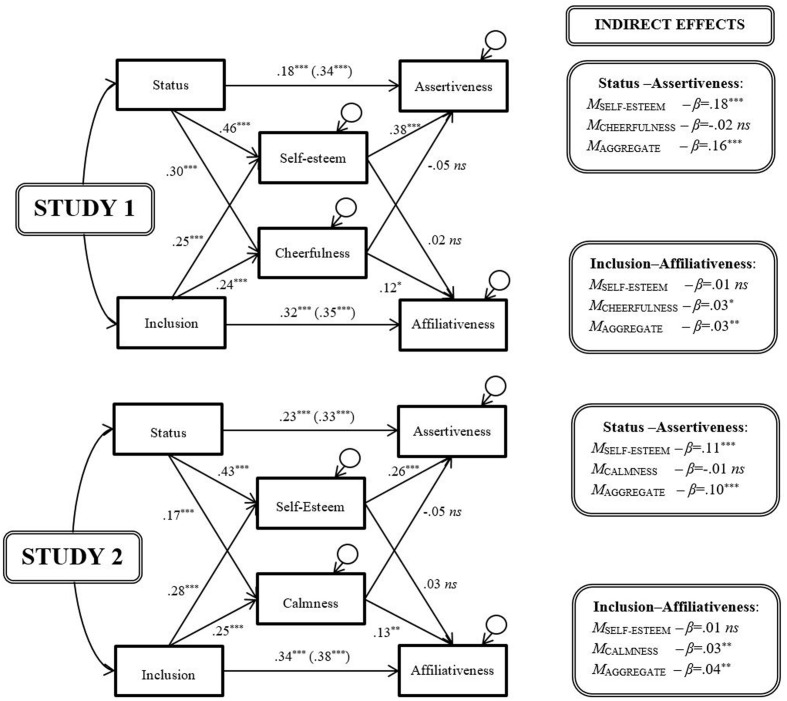
**The mediating effects of self-esteem and cheerfulness/calmness.** ^†^*p* < 0.10; ^∗^*p* < 0.05; ^∗∗^*p* < 0.01; ^∗∗∗^*p* < 0.001.

Accordingly, to determine whether self-esteem uniquely mediated the link between social relations and behavioral strategy—that is, to test whether the predictions of hierometer theory or sociometer theory held above and beyond the predictions of alternative theories—we ran a further pair of models parallel to the second set above, but replacing narcissism either with depression (assessed in Study 1: **Figure 4**, top) or anxiety (assessed in Study 2: **Figure 4**, middle). To facilitate inter-model comparison, we also inverted the depression and anxiety indices, such that higher scores represented lower depression (i.e., *cheerfulness*) and lower anxiety (i.e., *calmness*).

Compared to lone mediation by self-esteem (**Figure 2**), the impact of adding either cheerfulness or calmness to the models, as potential ancillary mediators, differed for the hierometer-relevant agentic variables and the sociometer-relevant communal variables. The path coefficients for status and assertiveness, direct and indirect, hardly changed. The same was true of the path between inclusion and self-esteem. However, the paths between self-esteem and affiliativeness changed substantially: they dropped to non-significance with the addition of either cheerfulness or calmness to the model, ruling out self-esteem serving any unique imperative function. Rather, the data in Study 1 were consistent with cheerfulness independently exhibiting a consolidatory dynamic—with higher inclusion prompting greater affiliativeness by increasing cheerfulness. Such indicative and imperative functions bear some similarity to those specified by evolutionary theories of clinical disorders (Price et al., 1994). In addition, a positive and significant path emerged between status and cheerfulness, although not between cheerfulness and assertive behavior—only partly in keeping with such theories. In a similar vein, the path coefficients in Study 2 suggested—again with self-esteem entered simultaneously in the model—that calmness exhibited a consolidatory dynamic like cheerfulness: higher inclusion prompted greater affiliativeness by increasing calmness. However, and again mimicking the pattern for cheerfulness, a positive and significant path emerged between status and calmness, although not between calmness and assertive behavior.

In sum, even when cheerfulness and calmness were included in the models as co-mediators, self-esteem continued to show, in near-unchanged form, the patterns of association with status and assertiveness predicted by hierometer theory. However, although self-esteem also continued to show associations suggestive of tracking inclusion, any associations suggestive of behavioral regulation disappeared. Yet, both cheerfulness (i.e., the inverse of depression) and calmness (i.e., the inverse of anxiety) showed the pattern consistent with the consolidatory dynamic previously shown by self-esteem, in line with evolutionary accounts of their adaptive function.

## General Discussion

In this article, we put forward, and empirically tested, a novel theory of the evolutionary function of self-regard: hierometer theory. This theory proposes that self-regard operates as part of a system that enables individuals to navigate status hierarchies adaptively. In doing so, individuals must make judicious decisions about whether or not to enter risky zero-sum contests that may not only be beneficially won but also harmfully lost. One factor liable to predict the outcome of such contests is prior social status. Accordingly, hierometer theory proposes that higher (lower) prior social status promotes a behavioral strategy of augmented (diminished) assertiveness, with self-regard acting as the intrapsychic bridge—in particular, tracking social status in the first instance and then regulating behavioral strategy in terms of it. Note that the overall dynamic involved is consolidatory rather than compensatory: higher rather than lower status is proposed to lead to increased assertiveness. In this regard, hierometer theory differs from dominance theory, which arguably implies that it is losses in social status that prompt attempts to regain it (Barkow, 1980).

Hierometer theory also supplements and complements sociometer theory, one leading theory of self-regard’s evolutionary function. In its original version, sociometer theory proposes that self-regard—in particular, self-esteem—operates as part of a system that enables people to maintain a minimal level of social acceptance, considered essential to survival. It proposes that self-esteem tracks levels of social acceptance, such that, if social rejection looms, a drop in self-esteem serves as an intrapsychic warning signal, motivating individuals to regain social acceptance by engaging in pro-social (i.e., affiliative) behavior. Note that the dynamic involved is compensatory: *Lower* rather than higher acceptance is proposed to lead to increased affiliativeness. A revised version of sociometer theory instead proposes that self-esteem tracks relational value more generally; that is, how much one perceives that others value the relationships that one has with them (Leary, 2005).

The explicit formulation of hierometer theory brings out a number of unresolved issues with sociometer theory. The most important of these has to do with the ambiguity over what “social acceptance” means, and by extension, over what “relational value” means.

In particular, individuals can become valued members of society in one of two ways: (a) by being insiders rather than outsiders—that is, by being liked, accepted, and included (Leary et al., 1995); or (b) by being winners rather than losers— that is, by being respected, admired, and deemed important (Magee and Galinsky, 2008). Otherwise put, individuals can achieve social inclusion or social status—two conceptually distinct, if empirically correlated, constructs. The question then arises as to whether and what extent self-regard tracks one versus the other—the former being more communal in character, and the latter being more agentic (Bakan, 1966; Huo et al., 2010). Whereas hierometer theory proposes that self-regard tracks status, the original version of sociometer theory proposes that self-regard (self-esteem) tracks inclusion, whereas the revised version leaves the issue open (i.e., tracks any form of relational value). Moreover, both hierometer theory and sociometer theory propose, not only that self-regard tracks social relations, but also that it regulates behavior. Specifically, whereas hierometer theory proposes that self-regard regulates assertive behavior, the original version of sociometer theory proposes that it (self-esteem) regulates affiliative behavior, with the revised version again leaving the issue open (i.e., regulates any behavior relevant to relational value).

Accordingly, by (a) taking the agentic constructs specified by hierometer theory, and the communal constructs specified by the original version of sociometer theory, and by (b) operationalizing them systematically and simultaneously at appropriate levels of analysis (i.e., social, psychological, behavioral), it becomes possible to test, via correlational and mediational means, and controlling for overlapping variance between parallel variables, whether and to what extent self-regard plays the indicative and imperative functions specified by both theories. It also becomes possible to test whether the dynamic involved is either compensatory (such that lower self-regard, evoked by lesser status or inclusion, prompts greater assertiveness or affiliativeness) or consolidatory (such that higher self-regard, evoked by greater status or inclusion, prompts greater assertiveness or affiliativeness). In addition, it is possible to test all the above with respect to two types of self-regard (self-esteem and narcissism), and also controlling for potential confounding variables of a clinical sort (depression and anxiety). In the research reported in this article, we actualized all these possibilities.

At every turn, the predictions of hierometer theory were verified. In both studies, we found that self-regard, whether in the form of self-esteem or narcissism, correlated positively with both status and assertiveness—consistent with self-regard tracking the former and regulating the latter. In addition, self-regard statistically mediated the link between status and assertiveness—partially for self-esteem alone, but wholly for self-esteem and narcissism combined, thereby highlighting the value of considering both forms of self-regard jointly, rather than self-esteem alone. The pattern of observed statistical associations suggested that higher (lower) status increases (decreases) self-regard which in turn augments (diminishes) assertiveness. This suggests a consolidatory dynamic: those who have status adopt a riskier behavioral strategy, and those who lack it adopt a safer one, with self-regard acting as the intrapsychic bridge. Furthermore, even when controlling for depression and anxiety—clinical variables also hypothesized to serve an evolutionary role in moderating levels of assertiveness—the correlations consistent with the hypothesized indicative and imperative functions remained robust, thereby raising the likelihood that self-regard indeed acts as the crucial mediator.

Second, the predictions of the original version of sociometer theory were sometimes verified and sometimes falsified, such that, on the whole, the theory was not supported (subject to some caveats below). In particular, self-esteem correlated positively with both inclusion and affiliativeness—consistent with it tracking the former and regulating the latter. Moreover, when considered in isolation, self-esteem also statistically mediated the link between the two. However, the pattern observed suggested that higher (lower) inclusion increases (decreases) self-esteem so as to augment (diminish) affiliativeness. This would imply an indicative function in keeping with the original version of sociometer theory, but an imperative function at odds with it (i.e., decreased self-esteem should augment affiliativeness). Furthermore, when competing clinical variables (i.e., depression and anxiety) were entered into models alongside self-esteem, all links to affiliativeness approached zero, thereby casting doubt on whether self-esteem is an independent mediator of any dynamic, consolidatory or compensatory. Finally, although narcissism did correlate negatively with affiliativeness, it barely correlated with inclusion; hence, as an alternative index of self-regard, narcissism cannot play the role required by self-regard in the original version of sociometer theory either. Note that our findings here are generally in keeping with the accumulated empirical record on sociometer theory to date, which reliably supports the hypothesized indicative function for self-esteem (Leary et al., 1995; Denissen et al., 2008), but only equivocally supports its hypothesized imperative function (Maner et al., 2007; Twenge et al., 2007).

That our findings for self-esteem were consistent with hierometer theory, but not or not entirely with the original version of sociometer theory, further suggests that, although self-esteem is both agency-based and communion-based, it is relatively more agency-based than communion-based—a conclusion also reached independently by other researchers (Wojciszke et al., 2011). Indeed, in this connection, Gebauer et al. (2015) recently found, in two very large cross-cultural studies, featuring self-reports and informant reports respectively, that the Big Five personality trait of extraversion—the one most related to agency and “getting-ahead”—uniquely predicted self-esteem, whereas the trait of agreeableness—the one most related to communion and “getting along”—did not. In addition, and as expected, narcissism emerged as relatively more agency-based than communion-based (Sedikides et al., 2002; cf. Campbell and Foster, 2007), in that it entailed a deficit in communion for some links (e.g., to affiliativeness) and a surfeit in agency for others (e.g., to assertiveness). Nonetheless, some links (e.g., to status) were equally strong for self-esteem and narcissism.

We believe that the foregoing discussion illustrates how our correlational and mediational findings—by being either in keeping, or at odds, with different theoretically derived predictions—serve either to provide support for, or to call into question, the theories that yield those predictions, thereby moving forward the investigation of the function of self-regard. That is, even if correlation and mediation do not prove causation, causal theories can nonetheless be tested by whether or not observed patterns of correlation and mediation are consistent with their predictions. In other words, although consistent patterns do not decisively establish a theory, inconsistent patterns do have the power to undermine it. As one of the progenitors of sociometer theory has emphasized “[absent] measurement error or methodological shortcomings … null correlations falsify causal hypotheses (Baumeister et al., 2003, p. 9).” For example, in the current research, a failure to find a unique positive correlation between status and self-esteem, after controlling for both inclusion and narcissism, would have called hierometer theory into question, and unequivocally supported the original version of sociometer theory, which emphasizes inclusion. Especially as a first step toward validating a promising theory, such correlational and mediational patterns are, at the very least, informative. In any case, complementary experimental research is now underway that has begun to establish the causal links specified by hierometer theory (Mahadevan et al., 2015).

In defense of sociometer theory, however, at least two caveats merit consideration. First, we focused on *long-standing* conditions, at a social, psychological, or behavioral level. Accordingly, we assessed all our variables in either chronic or trait form. Had we instead focused on *changing* conditions, and so assessed our variables in either acute or state form, more evidence of a compensatory dynamic might have come to light. Specifically, in line with the original version of sociometer theory, acute drops in state self-esteem might have momentarily augmented affiliativeness (Maner et al., 2007). Similarly, in line with dominance theory, acute drops in self-regard might have momentarily augmented assertiveness (Barkow, 1980). Ultimately, the timespan over which relevant variables are measured may be a critical moderator of, and a limiting condition on, the applicability of hierometer and sociometer theory. Nonetheless, given that links between constructs assessed at a chronic and trait level have been previously adduced as evidence for sociometer theory (Leary et al., 1995, 2001; Anthony et al., 2007a; Denissen et al., 2008), it is legitimate that they be adduced as evidence for hierometer theory, especially given its novelty. To count such chronic links as evidence for sociometer theory, but not for hierometer theory, would be tantamount to discrimination.

Second, our findings are arguably consistent with the *revised* version of sociometer theory, which is equivocal about the type of relational value that self-esteem tracks, and by extension, the type of social acceptance that goes hand in hand with it. Indeed, hierometer theory, and the original version of sociometer theory, might each be considered complementary subsets of the revised version of sociometer theory, if the latter is construed very broadly as a theory which states that types of social relations (status, inclusion), which constitute different types of relational value, regulate types of behavioral strategies (assertiveness, affiliativeness) via types of self-regard (self-esteem, narcissism). If so, then our confirmatory findings for hierometer theory, and mixed findings for the original version of sociometer theory, would still suggest that the revised version of sociometer theory holds truer for agentic variables than for communal ones.

## Conclusion

It matters whether, in the social world, one is either a winner or a loser, or an insider or outsider. Both outcomes seem to independently shape how individuals feel about themselves, and in turn, how they behave. But the pattern of data we obtained is most consistent with the outcome of being a winner or loser ultimately regulating how assertively or acquiescently individuals behave. In particular, prompted by their levels self-regard—people on an already winning trajectory seem motivated to seek further wins, whereas those on a losing trajectory seem motivated to avoid further losses. Such a dynamic system, which serves to consolidate individuals’ existing statuses, arguably helps them prudently navigate social hierarchies, by optimizing their judicious participation in risky zero-sum contests.

## Author Contributions

The theory was developed by NM and AG, with input from CS and WDW-A. NM and AG developed the studies and NM collected the data. Data were analysed by NM (primary) and AG (secondary). NM and AG co-wrote the manuscript which was then further refined by CS, WDW-A, NM, and AG.

## Conflict of Interest Statement

The authors declare that the research was conducted in the absence of any commercial or financial relationships that could be construed as a potential conflict of interest.

## Footnotes

1 The terms *self-regard* and *self-esteem* are often used interchangeably. Here, we use the term self-regard as an umbrella term to denote any global evaluation of the self, and the terms *self-esteem* and *narcissism* to refer to two types of self-regard. We are not the first to characterize self-esteem and narcissism as alternative ways of evaluating the self; several other rxesearchers have also made this distinction (Baumeister et al., 2003; Kernis, 2003; Paulhus et al., 2004). We elaborate on these distinctions later, but mention them immediately to pre-empt confusion. To anticipate one of our key contentions: we shall argue on theoretical grounds that self-esteem and narcissism serve different functional roles in social life.

2 Given that Gebauer et al. (2013) found a stronger correlation between self-esteem and self-ascribed agentic traits (e.g., ambitious) for men, and between self-esteem and self-ascribed communal traits (e.g., caring) for women, we also tested whether gender moderated the suite of effects we report below. In general, equivalently significant and substantial effects were obtained for both genders. Given the exposition is already lengthy, interested readers may contact the authors for further details.

3 Given the multifactorial nature of the NPI-40 in Study 2, we also tested whether the effects we report below obtained similarly or differently for the three key facets of narcissism isolated by Ackerman et al. (2011), namely, leadership/authority, grandiosity/exhibitionism, and exploitativeness/entitlement (see also Lee et al., 2013). Although effects obtained for overall narcissism ultimately replicated across all facets, exploitativeness/entitlement was less strongly related to agentic variables (e.g., status, assertiveness), and more negatively related to communal variables (e.g., inclusion, affiliativeness). Again, given the length of the exposition, interested readers are encouraged to contact the authors for further details. The unifactorial nature of the NPI-16 obviated any such supplementary analysis in Study 1.

4 Note that, in principle, many other mediational models could have been considered, some more piecemeal (e.g., involving only hierometer-relevant variables), and others more comprehensive (e.g., involving ancillary variables such as depression and anxiety). However, space limitations regrettably forbade us from considering all of them. We took the defensible view that self-esteem, the canonical form of self-regard invoked by both hierometer theory and sociometer theory, should be tested as the primary mediator of links between social relations and behavioral strategy, and that narcissism, a variant form of self-regard, should be tested as an ancillary mediator.

5 In the graphical representations of each model, two direct effects and error-covariances are not displayed for ease of presentation; however, these were included in the statistical models for purposes of estimation.
